# Supplementary material for: Leptin-Mediated Induction of IL-6 Expression in Hofbauer Cells Contributes to Preeclampsia Pathogenesis
Source: Int J Mol Sci. 2023 Dec 21;25(1):135. doi: 10.3390/ijms25010135 (PMC10778808; doi:10.3390/ijms25010135)
Supplement: Supplementary file 1 [file ijms-25-00135-s001.zip › ijms-2699847-supplementary.pdf]

Supplementary Materials:

Supplementary Figure S1.

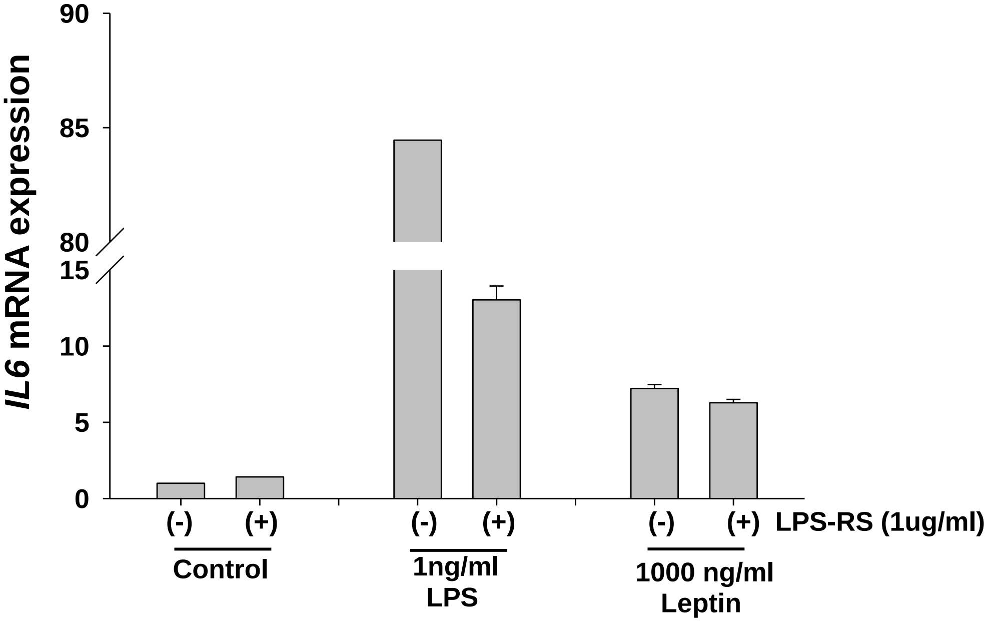

Supplementary Figure S1. Evaluation of the effect of residual endotoxin contamination of r-leptin on IL-6 expression. *IL-6* mRNA expression in primary cultured Hofbauer cells that were pre-incubated with 1μg/ml LPS antagonist (LPS-RS) for 1h, then incubated with either 1ng/ml LPS or 1000 ng/ml r-leptin for 6 h. LPS-RS treatment downregulated LPS-induced IL-6 mRNA levels, but no effect on r-leptin-induced IL-6 mRNA levels. Bars represent mean± SEM, n=2 repeat from one cultured Hofbauer cells.

Supplementary Figure S2.

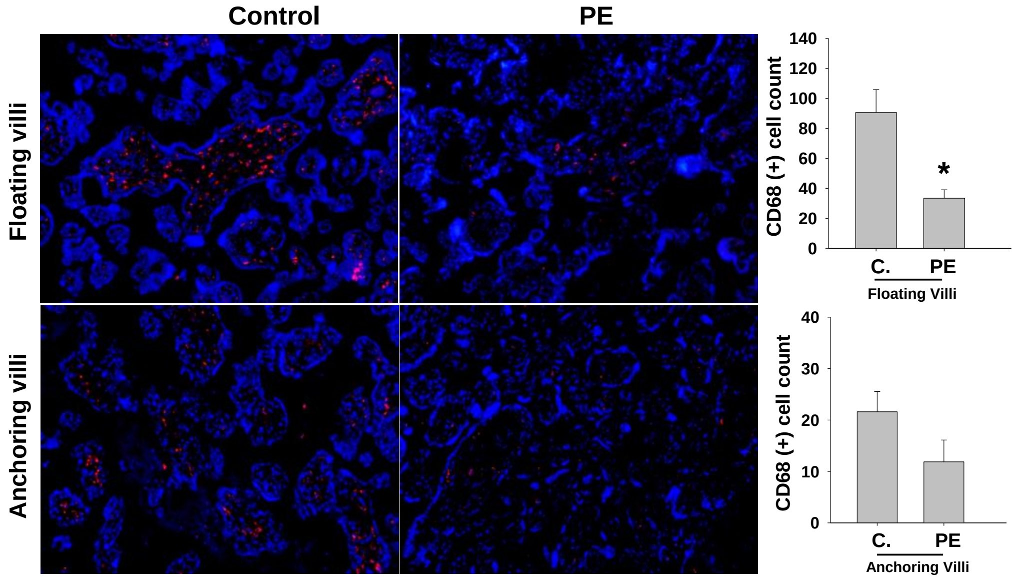

Supplementary Figure S2. CD68 (+) HBCs in control vs. PE placentas. Immunohistochemistry revealed a smaller number of CD68(+) cells both in floating villi (33.40 ± 5.63 vs. 90.53± 15.28, $p<0.05$) and anchoring villi (11.87± 4.24 vs. 21.60± 3.94, $p=0.131$) of PE placentas compared to their GA- match controls. Red:CD68 (+) staining, blue: DAPI counterstaining. Magnification 20x. PE: Preeclampsia, Bars represent mean± SEM, n=5, *: $p<0.05$ analyzed by Mann Whitney Rank Sum test.
